# Screening of mutations in NOL3 in a myoclonic syndromes series

**DOI:** 10.1007/s00415-014-7463-z

**Published:** 2014-08-20

**Authors:** A. Macerollo, N. E. Mencacci, R. Erro, C. Cordivari, M. J. Edwards, N. W. Wood, Kailash P. Bhatia

**Affiliations:** 1 Sobell Department of Motor Neuroscience and Movement Disorders, Institute of Neurology, University College London, London, UK; 2 Department of Molecular Neuroscience, Institute of Neurology, University College London, London, UK

Dear Sirs,

Recently, Russel and colleagues [1] performed a detailed genetic analysis of a four-generation family with a phenotype characterized by autosomal dominant, adult onset, cortical myoclonus without associated seizures. The authors proposed the term familial cortical myoclonus (FCM).

They demonstrated linkage to chromosome 16q21-22.1 and identified the E21Q missense change in *NOL3* (nucleolar protein 3) as the only segregating novel variant [1].

The NOL3 gene encodes a 208 amino acid protein that is expressed in heart, skeletal muscle, and brain. The amino acid A80, involved by the mutation we identified, resides in the N-terminal CARD, a motif that mediates protein–protein binding via electrostatic interactions. One possibility is that the A80T variant, similarly to what was hypothesized for the E21Q described by Russel and colleagues, may alter the electrostatic surface potential of the NOL3 CARD domain and change its binding properties for interaction with protein partners. Given the absence of an excitability phenotype in NOL3 knock-out mice and the presence of heterozygous loss-of-function variants in neurologically normal human control subject, it seems plausible that pathogenic NOL3 mutations may cause neuronal hyper-excitability through a gain-of-function mechanism [2, 3].

To investigate the contribution of the mutations in *NOL3* gene in myoclonic patients of British origin, we analyzed the complete coding sequence of *NOL3* in a cohort of 107 subjects (features summarized in Table 1).

**Table 1:** Demographics and clinical diagnoses of all patients

Type of myoclonus syndrome	Number of patients (sex ratio: male/female)	Age^a^	Age at onset^a^
Myoclonus-dystonia-like			
• Familial cases	11 (6/5)	49 ± 15	28 ± 18
• Sporadic case	86 (37/49)	50 ± 17	27 ± 15
Cortical myoclonus			
• Familial cases	3 (1/2)	46 ± 8	24 ± 6
• Sporadic case	7 (2/5)	59 ± 9	22 ± 4
Total	107 (46/61)	50 ± 16	27 ± 15

^a^Mean age in year ± standard deviation (SD)

Cases were selected where they were affected with cortical myoclonus or with either isolated subcortical myoclonus (previously known as essential myoclonus) or a combination of myoclonus and dystonia. We excluded patients who were positive for mutations in the *SGCE*, the most common genetic cause of myoclonus-dystonia [4]. Secondary forms of myoclonus had been excluded with the appropriate investigations.

We included three unrelated cases of autosomal dominant FCM with onset in the beginning of the third decade. In all patients cortical myoclonus was not associated with epilepsy. These patients underwent somatosensory evoked potentials (SSEPs) studies, which showed clear giant evoked potentials [5].

Furthermore, we included seven cases of sporadic cortical myoclonus without seizures. Myoclonus was triggered by action or inadvertent somatosensory stimuli. Video EEG–EMG recording with back-averaging analysis was performed in all patients, confirming the cortical origin of the myoclonus. Three of these seven patients also underwent SSEPs studies, which disclosed enlarged responses. Secondly, to explore the potential role of *NOL3* in myoclonus-related phenotype, we studied 97 patients affected by either familiar or sporadic undiagnosed myoclonus-dystonia-like syndrome. Our cohort included 11 familial cases with autosomal dominant pattern of inheritance and 86 sporadic cases.

In the 107 patients screened, we identified only one novel single nucleotide mutation in *NOL3* (c.238G>A; p.A80T) in one subject. This variant was absent in the 1000 genome project (http://www.1000genomes.org/) and Exome Variant Server (http://evs.gs.washington.edu/EVS/) databases. It is predicted to be damaging by Polyphen-2 (HumVar-trained http://genetics.bwh.harvard.edu/pph2/) and Mutation Taster (http://www.mutationtaster.org/) but tolerated by SIFT (http://sift.jcvi.org/). The amino acid residue involved by the missense change is conserved through species.

The mutation was identified in a male patient who exhibited myoclonic jerks affecting the face and both arms (right > left) that started in the fourth decade. The abnormal movements often developed into fast rhythmic clusters of jerks, but they did not show stimulus sensitivity. There was no family history of such conditions. The EEG did not show cortical correlates of myoclonic jerks. Furthermore, he was affected by epilepsy characterized by generalized seizures since age 15 for which he was on Sodium Valporate.

No other possibly pathogenic variants were identified.

The missense mutation p.E21Q, which was found to segregate in the pedigree described by Russel et al. [1], was not identified in our cohort. Functional work proving the functional effect whereby the identified *NOL3* change could lead to membrane hyperexcitability and cortical myoclonus is still lacking. This leaves open the possibility that the detected variant could be a private mutation or a very rare benign variant linked to the actual disease mutation but not itself pathogenic [6].

However, we have identified a new variant, p.A80T, in one patient of our cohort. Notably, the phenotype of our *NOL3* mutation carrier is different from what has been previously described. Indeed, our patient had subcortical myoclonus, seizures and no family history for such disorders. Unfortunately other family members were not available for clinical or genetic analysis, therefore the pathogenic role of the detected variant remains uncertain. Further reports of large series of patients suffering from either familial or sporadic cortical myoclonus or related phenotypes looking at *NOL3* variants are needed as well as functional studies on the gene function.
